# Radiographic angles in hallux valgus: Comparison between protractor and iPhone measurements

**DOI:** 10.1002/jor.22872

**Published:** 2015-03-30

**Authors:** Hong‐Zheng Meng, Wei‐Lin Zhang, Xiu‐Cheng Li, Mao‐Wei Yang

**Affiliations:** ^1^ Department of Orthopaedics First Affiliated Hospital China Medical University Shenyang 110001 PR China

**Keywords:** hallux valgus, radiographic angles, smartphone, measurement variability

## Abstract

Radiographic angles are used to assess the severity of hallux valgus deformity, make preoperative plans, evaluate outcomes after surgery, and compare results between different methods. Traditionally, hallux valgus angle (HVA) has been measured by using a protractor and a marker pen with hardcopy radiographs. The main objective of this study is to compare HVA measurements performed using a smartphone and a traditional protractor. The secondary objective was to compare the time taken between those two methods. Six observers measured major HVA on 20 radiographs of hallux valgus deformity with both a standard protractor and an Apple iPhone. Four of the observers repeated the measurements at least a week after the original measurements. The mean absolute difference between pairs of protractor and smartphone measurements was 3.2°. The 95% confidence intervals for intra‐observer variability were ±3.1° for the smartphone measurement and ±3.2° for the protractor method. The 95% confidence intervals for inter‐observer variability were ±9.1° for the smartphone measurement and ±9.6° for the protractor measurement. We conclude that the smartphone is equivalent to the protractor for the accuracy of HVA measurement. But, the time taken in smartphone measurement was also reduced. © 2015 The Authors. *Journal of Orthopaedic Research* Published by Wiley Periodicals, Inc. J Orthop Res 33:1250–1254, 2015.

Radiographic angles are used to assess the severity of hallux valgus deformity, select the type of surgical procedure^1^ for correction, and assess postoperative outcomes. The radiographic angles are commonly used in patients with hallux valgus angle (HVA), inter‐metatarsal angle (IMA), distal metatarsal articular angle (DMAA), or inter‐phalangeal angle (IPA). Traditionally, HVA is measured by using a protractor and a marker pen with hardcopy radiographs. But it has been shown that this method is error‐prone and time‐consuming.^2^, ^3^, ^4^, ^5^, ^6^

Now, measurements of digitized images using computer‐assisted image analysis software have been widely used, and these methods are more accurate and efficient.^7^, ^8^, ^9^ The picture archiving and communication systems (PACS) which provide economical storage of, and convenient access to, images from multiple modalities can directly measure radiographic angles using software, so the increased application of PACS has reduced the need for manual measurement of radiographic angles. But, just a few hospitals have this system in developing countries, so this technology is not universal. On the other hand, the digital measurement tools which the software based on are generally not portable or useful when the clinician is consulting with patients outside of the hospital or computer networked facility. For these reasons, hallux valgus measurements using hard copy radiograph films or printed versions of digital radiographs are still widely performed, so it is necessary to find a tool which is more convenient and more efficient to measure HVA.

The new generation of cellular phones—“smartphones”—makes angle measurement possible by using an integrated accelerometer, and provides a potentially useful clinical tool for measuring HVA.

The main objective of this study was to evaluate HVA measurements using a protractor and an smartphone. The secondary objective was to compare the time taken between those two methods.

## MATERIALS AND METHODS

### Selection of Radiographs

Twenty weight‐bearing radiographs for patients with hallux valgus deformity which have been diagnosed by the expert of orthopedics from an outpatient clinic of The First Hospital of China Medical University were used in this study. The weight‐bearing radiographs used in this study were taken according to the standards made by Smith RW et al.^10^ The 20 radiographs were printed on A4 paper.

### Observers

The radiographs were assessed by six orthopedic surgeons with areas of special interest in foot and ankle. All of them have trained for 1 week so as to be familiar with the software that used to measure the HVA.

### Angles

Due to the methods and steps being identical, we chose HVA as an example of HVAs (HVA, IMA, DMAA, IPA) to complete this study.

### Radiographic Measurements

#### Protractors

There are many described methods^11^, ^12^, ^13^, ^14^ to measure these HVAs using a protractor and a marker pen, but there was no particular advantage to any measuring method.^15^ And also, there is no universally accepted method. In this study, we use the method described by Venning and Hardy^14^ to measure these angles. The widths of each metatarsal and proximal phalanx are halved at two levels; the points are joined and extended in both directions to draw the axes.

#### Smartphones

**Figure 1:**
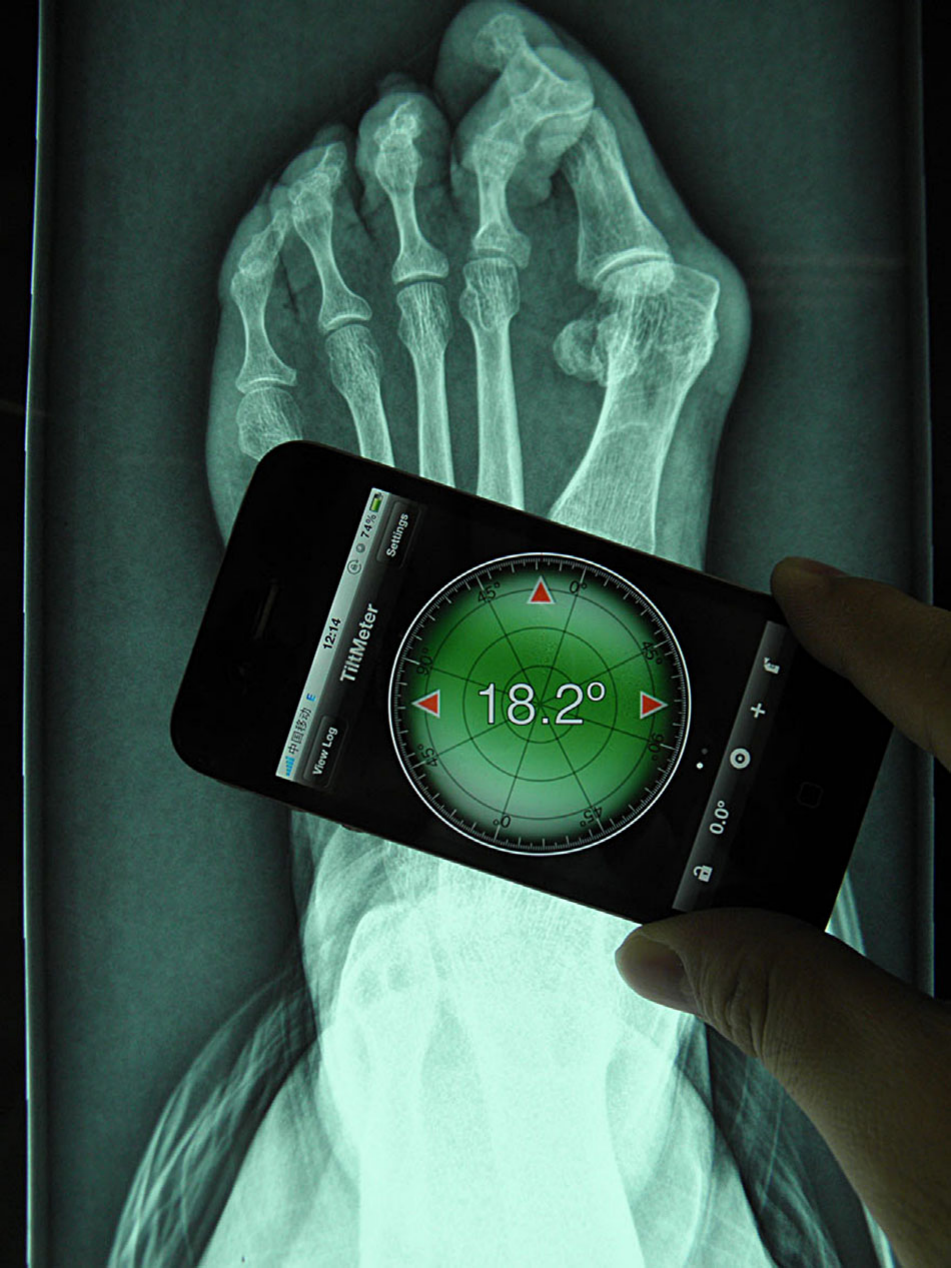
Measuring hallux valgus angle (HVA) by smartphone.

We performed all the smartphone measurements with an Apple iPhone (Apple, Inc, Cupertino, CA) running the Tiltmeter software, which was downloaded from the Apple iTunes store. Measurement of HVA using the smartphone included measuring the angles of the first metatarsal and the first proximal phalanx. Those numbers were then added together to obtain the HVA (Fig. 1).

### Collection of Data

The measure order of the six observers was as follows:

Week 1: The six observers measured the HVA using smartphones.

Week 2: The six observers measured the HVA using protractors.

Week 3: Four observers randomly selected from the original six measured the HVA using smartphones again.

Week 4: The four observers who were selected last week measured the HVA using protractors.

The time of each measurement was recorded by the same person with a stopwatch. All the measurements and times were recorded on Excel sheets electronically by a fixed recorder. A total of 400 measurements were recorded. None of the observers had the right to see the results (both measurements they had made and measurements of other observers).

### Analysis of Data

The two HVA measurement methods were compared by using the method described by Bland and Altman.^16^, ^17^ Intra‐observer variability was assessed by analyzing the absolute difference between successive HVA angle (a) measurements using the same measurement tool by the same observer.

$a=\left|a_{n}-a_{n+1}\right|$, where n and n+1 are successive measurements. 95% confidence intervals for intra‐observer variability were calculated as ($\bar{x}$ ± t0.05(n−1) S / $\sqrt{n}$), where S is the standard deviation of the intra‐observer differences △a. The inter‐observer variability (standard deviation of the difference between measurements by two different observers) was calculated as ($\sqrt{2}$ ×S) for a single measurement per observer, where S is the standard deviation of the inter‐observer differences. The 95% confidence intervals for inter‐observer variability were calculated using 2.09×SD (t‐distribution with 19‐degree of freedom).

## RESULTS

### Demographics

The study group comprised of 20 females with an average age of 42 years (range 20–62 years). Assessments of the 20 radiographs by six observers and repeat assessments by four of the six observers, gave a total of 200 sets of measurements. The overall mean HVA for the group was 37° (range 21–53°). The mean measurement time for an observer to measure one HVA was 34.1 s (range 26.8–46.3 s) in the smartphone group and 55.4 s (range 43.3–68.4 s) in the protractor group, respectively (Table 1).

**Table 1:** Demographics

	Mean ± SD	Range
Age (years)	42 ± 15.3	(20–62)
HVA (°)	37 ± 12.9	(21–53)
t1^a^ (seconds)	34.1 ± 5.3	(26.8–46.3)
t2^b^ (seconds)	55.4 ± 4.8	(43.3–68.4)

^a^ The mean measurement time for an observer to measure one HVA in the smartphone group.

^b^ The mean measurement time for an observer to measure one HVA in the protractor group.

### Protractor Comparison Versus Smartphone

**Figure 2:**
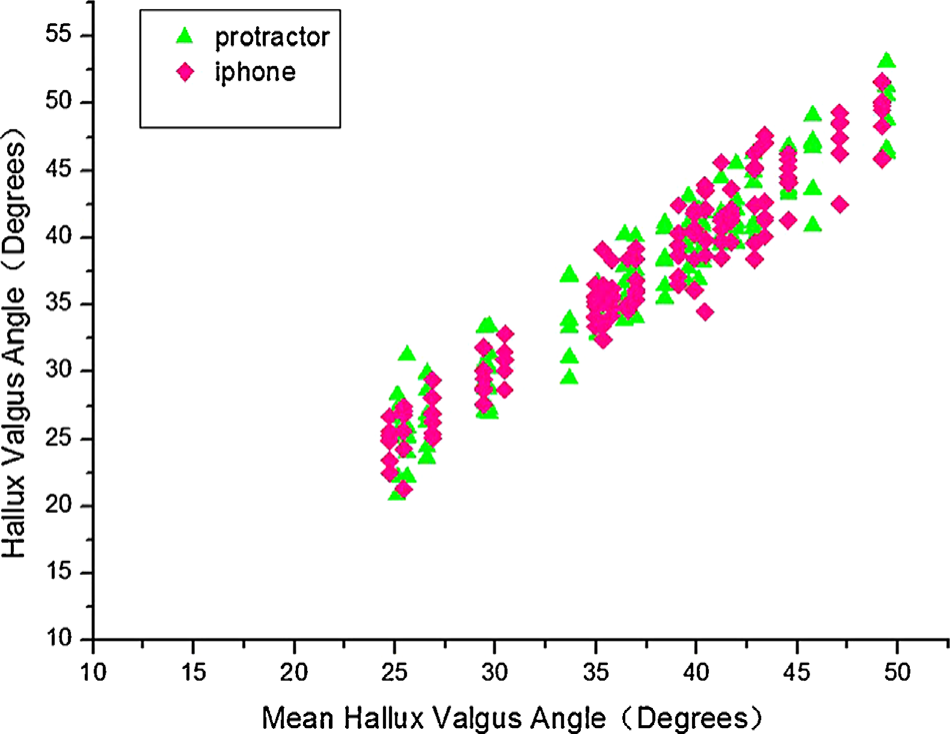
Scatter plot of all hallux valgus angle (HVA) measurements for both protractor and smartphone, plotted versus mean HVA for the radiograph.

**Figure 3:**
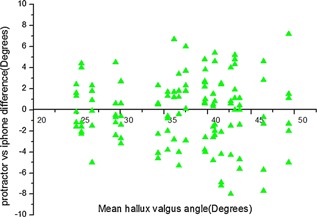
The difference of measurements measured by the same observer on the same radiograph using different methods.

Comparison of protractor and smartphone measurements (Fig. 2) shows all data points for both the protractor and smartphone measurements plotted against the mean HVA for each pair of the measurements. Figure 3 shows a graph of signed measurement difference between pairs of protractor/smartphone measurements for the same radiograph against mean HVA. The mean absolute difference between pairs of protractor and smartphone measurements was 3.2° (range 0–8°). The 95% confidence interval for differences between protractor and smartphone measurements on the same radiograph was ±3.7°, suggesting that there is a small measurement bias between smartphone and protractor measurements and the bias has not a clinically significant difference in HVA angle.

### Intra‐Observer Variability

**Figure 4:**
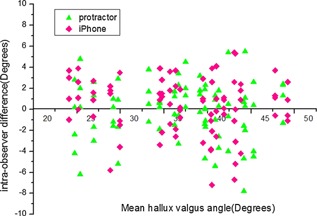
The difference of measurements measured by the four observers who randomly selected in week 3 for the same radiograph using same tool.

Figure 4 shows the difference between pairs of successive measurements by the same observer for both the protractor and smartphone measurements, plotted against mean HVA. The mean absolute intra‐observer difference was 2.70° (range 0–7.2°) for the smartphone measurement and 2.71° (range 0–7.8°) for the protractor measurement. 95% confidence intervals for intra‐observer variability were ±3.1° for the smartphone measurement and ±3.2° for the protractor measurement (Table 2), suggesting that the intra‐observer variability of the smartphone is equivalent to the protractor.

**Table 2:** Intra‐Observer and Inter‐Observer Variability of the Smartphone Measurement and Protractor Measurement

	Intra‐Observer Variability^a^		Inter‐Observer Variability^b^	
	MAD^c^	95%CI	STD^d^	95%CI
Smartphone	2.70°	±3.1°	4.34°	±9.1°
Protractor	2.71°	±3.2°	4.60°	±9.6°

^a^ Assessed by analyzing the absolute difference between successive HVA angle measurements using the same measurement tool by the same observer.^16^, ^17^

^b^ Assessed by analyzing the standard deviation of the difference between measurements by two different observers.18

^c^ The mean absolute difference between pairs of protractor and smartphone.

^d^ The measurements standard deviation of the difference between measurements by two different observers.

### Inter‐Observer Variability

Based on a single reading by each observer, the S of a HVA measurement was 3.25° for the protractor measurement and 3.07° for the smartphone measurement. The inter‐observer error (standard deviation of the difference between measurements by two different observers) is, therefore, $\sqrt{2}$ ×S = 4.60 for the protractor measurement and 4.34 for the smartphone measurement. The 95% confidence intervals for inter‐observer error were ±9.60° and ±9.07° for the protractor and smartphone measurements, respectively, (Table 2), suggesting that the inter‐observer variability of the smartphone is equivalent to the protractor.

### Time Consuming Comparison

**Figure 5:**
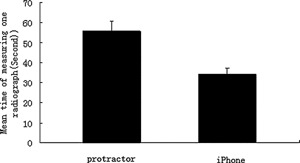
The mean time of measuring one radiograph by protractors or smartphones.

Figure 5 showing the mean time of measuring one radiograph was 55.4 s for the protractor group and 34.1 s for the smartphone group. Smartphone measurement was 40% faster than the protractor measurement.

## DISCUSSION

The increasing popularity of smartphones equipped with accelerometer has provided a new technology for electronic angle measurement. The nature of these devices and the easy access to application software mean that they may have a significant impact on efficiency and convenience of clinical assessment of hallux valgus deformities. We compared the performance of the smartphone with the protractor for measuring HVA. Sometimes, manual measurement of HVA using a protractor is replaced by software‐based measurements of digital radiographs, which is available through hospital PACS systems. However, not every hospital has been equipped with hospital PACS systems. Many clinicians may run clinics in rural areas where there is no digital radio‐graphic technology. Physicians who want to discuss the patient's condition may have radiographs emailed or mailed from distant locations and are unable to access the digital radiographic angles tools that are built into their hospital PACS system to measure these radiographs. Patients also may take the radiographs on computer that includes software without the digital radiographic angles tools. Smartphone offers a convenient tool to measure the radiographs, and smartphone can measure them directly on the computer screen, the printed paper, or from a hardcopy version of the radiograph. The mean time taken to measure the radiographs with the smartphone was over 40% less than that of protractor measurement, although this time, advantage would not be clinically significant for the measurement of a single radiograph. The measurement variability results indicate that the opacity of the smartphone did not increase HVA measurement variability, so we concluded that the observers performed their techniques successfully. In this study, due to the methods and steps being identical, we chose HVA as an example of HVAs (HVA, IMA, DMAA, IPA) to complete this study, and the result of this study is equally applicable to the other angles.

In this study, the mean absolute difference between pairs of protractor and smartphone measurements was 3.2°. It is less than the 5° difference which is widely accepted as signifying a clinically significant difference in HVA angle. Therefore, we conclude that the two measurements have no clinically significant difference in HVA angle. 95% confidence intervals for intra‐observer variability was ±3.1° for the smartphone group and ±3.2° for the protractor group. 95% confidence interval for inter‐observer variability was ±9.1° for the smartphone group and ±9.6° for protractor group. Therefore, we conclude that the smartphone, as a measuring tool, is better than the traditional protractor because of its convenience, efficiency, and mobility.

In addition to automatically adding the first metatarsal and the first proximal phalanx angles to obtain the HVA, newer versions of the Tiltmeter Pro software enable the user to store previous measurements, and thus allow the user to compare current readings with previous readings for a particular patient. Therefore, the smartphone could likely be used in many clinical measurement situations, for example, in the operating room. Traditionally, surgeons measure some important angles on the screen of C‐Arm or X‐ray machine by their eyes but now they can use smartphone which can give them an accurate number immediately. With the comparation of the key angles measured pre‐operatively and during the operation, the effect of the operation can be estimated timely and be used as a feedback to the surgeons that either gives them confidence to finish the operation perfectly or let them adjust the angle again to get a better angle.

In a conclusion, with the popularity of the smartphone, we believe that this method will be widely used in the clinical work.

## AUTHOR'S CONTRIBUTIONS

Meng HZ and Zhang WL contributed equally to this study. The authors have read and approved the final submitted manuscript, and have declared that no competing interests exist.
